# The Dietary Isoflavone Daidzein Reduces Expression of Pro-Inflammatory Genes through PPARα/γ and JNK Pathways in Adipocyte and Macrophage Co-Cultures

**DOI:** 10.1371/journal.pone.0149676

**Published:** 2016-02-22

**Authors:** Yuri Sakamoto, Junko Kanatsu, Mariko Toh, Ayano Naka, Kazuo Kondo, Kaoruko Iida

**Affiliations:** 1 Department of Nutrition and Food Science, Graduate School of Humanities and Sciences, Ochanomizu University, Otsuka, Bunkyo-ku, Tokyo, Japan; 2 Laboratory of Applied Nutrition, Faculty of Human Life and Environmental Sciences, Ochanomizu University, Tokyo, Japan; 3 Endowed Research Department “Food for Health”, Ochanomizu University, Tokyo, Japan; Nihon University School of Medicine, JAPAN

## Abstract

Obesity-induced inflammation caused by adipocyte-macrophage interactions plays a critical role in developing insulin resistance, and peroxisome proliferator-activated receptors (PPARs) regulate inflammatory gene expression in these cells. Recently, the soy isoflavone daidzein was reported to act as a PPAR activator. We examined whether daidzein affected adipocyte-macrophage crosstalk via the regulation of PPARs. Co-cultures of 3T3-L1 adipocytes and RAW264 macrophages, or palmitate-stimulated RAW264 macrophages were treated with daidzein in the presence or absence of specific inhibitors for PPARs: GW6471 (a PPARα antagonist), and GW9662 (a PPARγ antagonist). Inflammatory gene expression was then determined. Daidzein significantly decreased chemokine (C-C motif) ligand 2 (*Ccl2*, known in humans as monocyte chemo-attractant protein 1 (MCP1)) and interleukin 6 (*Il6*) mRNA levels induced by co-culture. In 3T3-L1 adipocytes, daidzein inversed the attenuation of adiponectin gene expression by co-culture, and these effects were inhibited by the PPAR-γ specific inhibitor. Daidzein also decreased *Ccl2* and *Il6* mRNA levels in RAW264 macrophages stimulated with palmitate or conditioned medium (CM) from hypertrophied 3T3-L1 adipocytes. This inhibitory effect on *Il6* expression was abrogated by a PPAR-α inhibitor. Additionally, we examined the activation of nuclear factor-kappa B (NF-κB) and c-Jun N-terminal kinase (JNK) pathways and found that daidzein significantly inhibited palmitate-induced phosphorylation of JNK. Our data suggest that daidzein regulates pro-inflammatory gene expression by activating PPAR-α and -γ and inhibiting the JNK pathway in adipocyte and macrophage co-cultures. These effects might be favorable in improving adipose inflammation, thus, treatment of daidzein may be a therapeutic strategy for chronic inflammation in obese adipose tissue.

## Introduction

Obesity is a worldwide concern and is associated with a state of chronic inflammation characterized by increased production of inflammatory cytokines/chemokines [1]. Several cell types, such as adipocytes and macrophages, are involved in cytokine production and induction of chronic inflammation [2]. In particular, the macrophages that are infiltrated in, and activated by, obese adipose tissue contribute to the elevation of inflammatory cytokines, such as tumor necrosis factor α (TNF-α), interleukin 6 (IL-6) and monocyte chemoattractant protein 1 (MCP-1, known as chemokine (C-C motif) ligand 2 (CCL2) in mice) [3–5]. These are attributed to systemic and local insulin resistance in an endocrine and paracrine fashion [6, 7]. Thus, chronic inflammation in adipose tissues is a key feature of obesity, and promotes the development of insulin resistance and Type 2 diabetes [8, 9].

Soy isoflavones are a group of polyphenolic compounds that have variety of biological actions [10–12]. To date, human and animal studies suggested that isoflavones play a beneficial role in improving glucose metabolism and insulin resistance and reducing obesity and diabetes [13, 14]. Although the precise mechanism is controversial, anti-inflammatory actions of isoflavones might be involved in the mechanism. Previous experimental evidence suggests that these polyphenols inhibit inflammatory changes via modulation of inflammatory signaling pathways thereby preventing a variety of common health disorders [15, 16]. In addition, it is reported that some isoflavones attenuate lipopolysaccharide (LPS)-induced inflammation via activation of the peroxisome proliferator-activated receptor (PPAR)-γ [17, 18].

PPARs are members of the nuclear receptor superfamily and three receptor s subtypes (PPAR-α, -β/δ and-γ) are expressed in mammals. PPARs, particularly PPAR-α and-γ, have emerged as key regulators in obesity-associated chronic inflammation in adipose tissue that contributes to insulin resistance [19, 20]. Given this, we previously reported that daidzein, a major isoflavone in soybeans, regulated cytokine expression both in adipose tissue of obese mice and cultured adipocytes through a PPAR-γ-dependent pathway, thereby lessening insulin resistance [21]. However, several previous studies suggested that PPAR-α is the dominant modulator for cytokine expression particularly in macrophages that have infiltrated in to adipose tissues [22, 23]. Moreover, although the suggested potential of some isoflavones as activators for PPAR-α has been reported [24], the anti-inflammatory effect of isoflavones on adipose tissue-resident macrophages, or PPAR-α involvement in their anti-inflammatory effect has not been investigated.

In the present study, we focused on daidzein and determined whether this compound alters the expression of pro-inflammatory cytokines in adipocyte- macrophage crosstalk through the regulation of PPARs. For this purpose, we used a co-culture model of adipocytes and macrophages, as an *in vitro* model of adipose inflammation.

## Materials and Methods

### Materials

Daidzein, GW6471 (an antagonist of PPAR-α), and GW9662 (an antagonist of PPAR-γ) were purchased from Cayman Chemical (Ann Arbor, MI, USA). Isobutylmethylxanthine, dexamethasone and insulin were purchased from Sigma-Aldrich (Tokyo, Japan).

### Cell culture

Murine 3T3-L1 preadipocytes (ATCC, Manassas, VA, USA), RAW264 macrophages and HEK293T cells (RIKEN, Tsukuba, Japan) were cultured in DMEM containing 10% fetal bovine serum (FBS) at 37°C in a humidified 5% CO_2_ atmosphere. Differentiation of 3T3-L1 preadipocytes was induced on day zero by the addition of 0.5 mmol/L isobutylmethylxanthine, 1 μmol/L dexamethasone and 10 μg/mL insulin. After 48 h (day 2), the medium was replaced with DMEM containing 10 μg/mL insulin and 10% FBS. The medium was changed every 2 days until the cells were used as differentiated 3T3-L1 at day 7 to 8 after the induction of differentiation. Hypertrophied 3T3-L1 with larger lipid droplets at day 21 were also used.

### Co-culture of adipocytes and macrophages

Co-culture of adipocytes and macrophages was performed using two different methods (S1 Fig) as previously described [7] but with some modifications. In the contact system, differentiated 3T3-L1 adipocytes were cultured in 6-well plates, and RAW264 macrophages (2.0 × 10^5^ cells/well) were plated onto 3T3-L1 adipocytes at day 7 in serum-free medium with or without 25 μM daidzein. The cells were cultured for 24 h and harvested. As a control, 3T3-L1 adipocytes and RAW264 macrophages, the numbers of which were equal to those in the co-culture, were cultured separately and mixed after harvest. In the transwell system, differentiated 3T3-L1 adipocytes were cultured in 6-well plates in the presence or absence of 25 μM daidzein, and RAW264 macrophages (2.0 × 10^5^ cells/well) were plated onto the transwell insert containing a 0.4 μm polyethylene terephthalate membrane (Greiner Bio-One, Tokyo, Japan) in serum-free medium with or without the indicated antagonist. After incubation for 24 h the, transwell was removed and 3T3-L1 adipocytes were harvested for analysis.

### Induction of inflammation in macrophages

Differentiation of 3T3-L1 adipocytes was conducted for 21 days. These hypertrophied 3T3-L1 adipocytes were cultured in serum-free medium for 12 h. Then the medium was collected as a conditioned medium (CM) and stored at −20°C until use. RAW264 macrophages were plated on 6-well plates at the cell density of approximately 4.0 × 10^5^ cells/well. Serum-starved RAW264 macrophages were treated with daidzein in the presence or absence of the indicated antagonist for 1 h, and thereafter incubated with CM from 3T3-L1 adipocytes for 24 h or media containing 400 μM palmitate for 4 h in the presence of daidzein with or without the antagonist.

### Transfection and luciferase assays

HEK293T cells were plated on 24-well plates at a cell density of approximately 2.5 × 10^4^ cells/well and were grown to 70–80% confluence. Cells were then transiently transfected with a PPAR-α or PPAR-γ expression plasmid, and a plasmid containing the luciferase gene under the control of three tandem PPAR response elements (PPRE × 3 TK-luciferase; gifts from Dr. Nakagawa, Tsukuba University, Japan) using an X-treme GENE HP DNA Transfection Reagent (Roche, Tokyo, Japan) according to the manufacturer’s protocol. Renilla luciferase control vectors were co-transfected to control for transfection efficiency. After transfection, cells were cultured for another 24 h in medium containing dimethyl sulfoxide (DMSO) or various concentrations (6.25, 12.5, 25 μM) of daidzein. Cells were lysed, and luciferase activity was measured and expressed as fold induction, that was normalized to the activity of the renilla luciferase control plasmid.

### RNA isolation and real-time PCR

Total RNA was isolated using Sepasol-RNA I reagent (Nacalai Tesque, Kyoto, Japan) according to the manufacturer’s protocol. First-strand cDNA was synthesized using a ReverTra Ace qPCR RT Master Mix (Toyobo, Osaka, Japan). Samples were run in 10 μL reactions using a SYBR^®^ Premix Ex Taq^™^ II (Tli RNaseH Plus) (Takara Bio, Shiga, Japan) on a 7300 real-time PCR instrument (Life Technologies Japan, Tokyo, Japan). Samples were incubated at 95°C for 30 s followed by 40 cycles at 95°C for 5 s and 60°C for 30 s. Genes were expressed as mRNA levels normalized to a standard housekeeping gene (β-actin) using the ΔΔCT method (S1 Table).

### Western blot analysis

RAW264 macrophages in serum-free medium were pretreated with or without 25 μM daidzein for 1 h, then stimulated with 400 μM palmitate for 4 h in the absence or presence of daidzein. Cells were washed with phosphate buffered saline (PBS) and then lysed in lysis buffer containing 10% Triton X-100, 50% glycerol, 5% sodium pyrophosphate, 1 M NaF, 100 mM Na_3_VO_4_, 10 × protease inhibitor, 20 mM HEPES pH 8, 25 mM NaCl, and incubated on ice for 15 min. The cellular lysate was centrifuged for 15 min at 12,000 rpm at 4°C. The supernatants were transferred into new tubes, and protein content was determined by the Bradford method using the Pierce Coomassie Protein Assay Kit (Life Technologies, Tokyo, Japan). Equal amounts of protein from all samples were separated on SDS-acrylamide gels (SDS-PAGE). After electrophoresis, proteins were transferred onto Hybond-P^™^ Blotting Membranes (GE Healthcare, Tokyo, Japan) and blocked with 5% BSA in Tris-buffered saline with 0.1% Tween 20 (TBS-T) at room temperature for 30 min. The membrane was incubated overnight at 4°C with specific primary antibody (S2 Table). After being washed with TBS-T three times, the blots were hybridized with secondary antibodies (S2 Table) conjugated with horseradish peroxidase for 30 min at room temperature. After being washed a further three times with TBS-T, the antibody-specific protein was visualized by the ECL detection system, ECL Prime, and the biomolecular imager ImageQuant LAS 4000 (GE Healthcare, Tokyo, Japan).

### Measurement of inflammatory mediators in culture supernatants

The amount of inflammatory mediators in culture supernatants was determined using commercially available ELISA kits for CCL2 and IL-6 (both from R&D Systems, Minneapolis, MN, USA) according to the manufacturers’ protocols. The values were normalized to the total protein content determined from cell lysates using Protein Assay CBB Solution (Nacalai Tesque, Kyoto, Japan).

### Statistical analysis

All experiments were performed at least twice independently and results expressed as means ± standard error (SE). An unpaired *t*-test (two groups) or a one-way factorial analysis of variance (three or more groups) was used for statistical analyses of experiments, as appropriate, using IBM SPSS Statistics software for Windows, Version 21.0. (IBM Corp., Armonk, NY, USA). Differences of *p*<0.05 were considered significant.

## Results

### Regulation of pro-inflammatory gene expression by daidzein in contact co-culture systems

**Fig 1:**
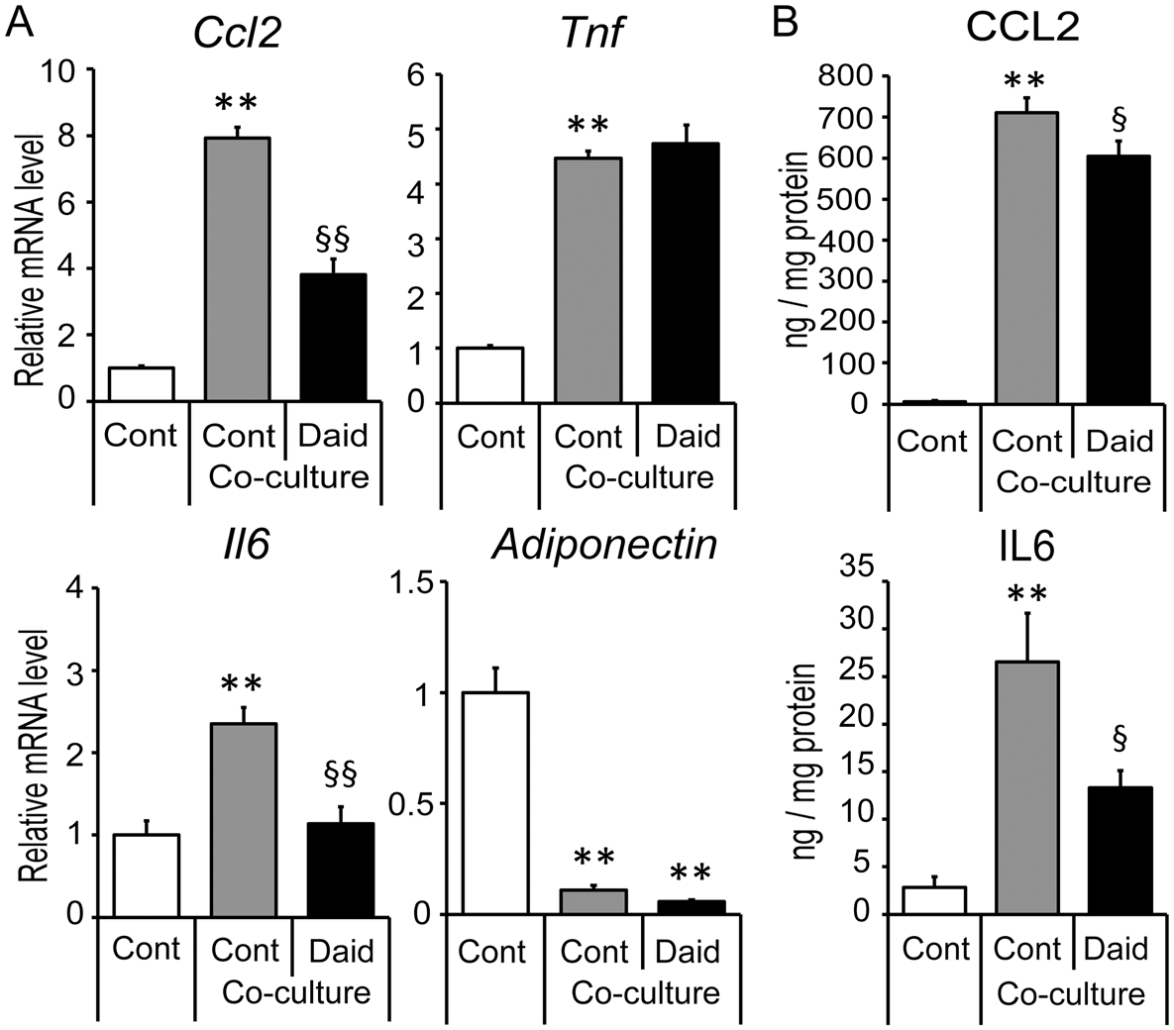
Effect of daidzein on pro-inflammatory gene expression in contact co-culture of 3T3-L1 adipocytes and RAW264 macrophages. 3T3-L1 preadipocytes were differentiated for seven days. RAW264 macrophages were co-cultured on adipocytes (Co-culture) in serum-free medium with (Daid) or without daidzein (Cont) for 24 h. (A) *Ccl2*, *Il6*, *Tnf* and adiponectin mRNA levels were quantified by real-time PCR and normalized to β-actin expression. Values are expressed as the fold change compared with a separate Cont culture arbitrarily set to 1. (B) The amounts of CCL-2, and IL-6 protein in the medium were determined by ELISA and normalized to the total cell protein content. **, *p*<0.01 versus Cont; §, *p*<0.05; §§, *p*<0.01 versus co-culture Cont.

**Fig 2:**
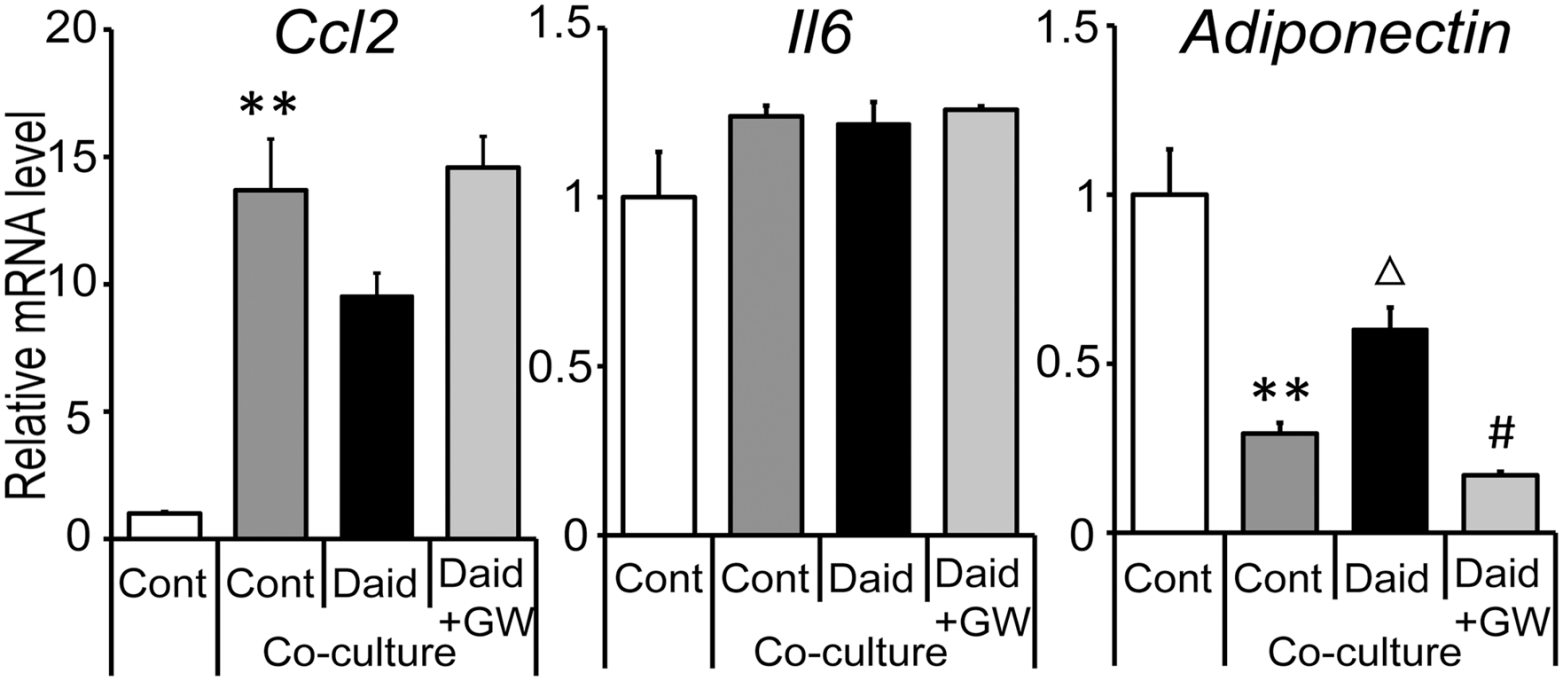
Effect of daidzein via PPAR-γ inhibition on pro-inflammatory gene expression in transwell co-cultured adipocytes. 3T3-L1 preadipocytes were differentiated for seven days. RAW264 macrophages were plated onto the transwell insert for 24 h. 3T3-L1 cells were differentiated in DMSO (Cont), 25 μM of daidzein (Daid), or Daid + 10 μM of GW9662 (Daid+GW). *Ccl2*, *Il6* and adiponectin mRNA levels in 3T3-L1 adipocytes were quantified by real-time PCR and normalized to β-actin expression. Values are expressed as the fold change compared with a Cont culture without macrophages arbitrarily set to 1. **, *p*<0.01 versus Cont; Δ, *p* <0.1 versus co-culture Cont; #, *p*<0.05 versus co-culture Daid.

We first examined the effects of daidzein in a contact co-culture system of 3T3-L1 adipocytes and RAW264 macrophages. The expression of inflammatory genes (*Ccl2*, *Il6* and *Tnf*; encoding CCL-2, IL-6, and TNF-α, respectively) was enhanced by co-culture as previously reported [7]. Daidzein treatment significantly inhibited the increase of *Ccl2* and *Il6* (Fig 1); in contrast, the expressions of *Tnf* and adiponectin (an anti-inflammatory cytokine secreted from adipose) were unaltered (Fig 1A). Consistent with this regulation, daidzein reduced the protein levels of MCP-1 and IL-6 released into the cell culture media (Fig 1B). Our previous study showed that daidzein decreased CCL2 and increased adiponectin at the gene and protein level in adipocytes [21]. Therefore, we next assessed whether its effect was also seen in co-cultured adipocytes using a transwell co-culture system. Daidzein attenuated the reduction of adiponectin expression in adipocytes, and a PPAR-γ specific inhibitor abrogated this effect. However, *Ccl2* and *Il6* expression were not changed by daidzein in co-cultured 3T3-L1 adipocytes (Fig 2), suggesting that daidzein mediated the macrophage-dependent decrease of *Ccl2* and *Il6* expression. *Tnf* expression was barely detected in this setting (data not shown).

### Activation of PPAR-α and PPAR-γ by daidzein

**Fig 3:**
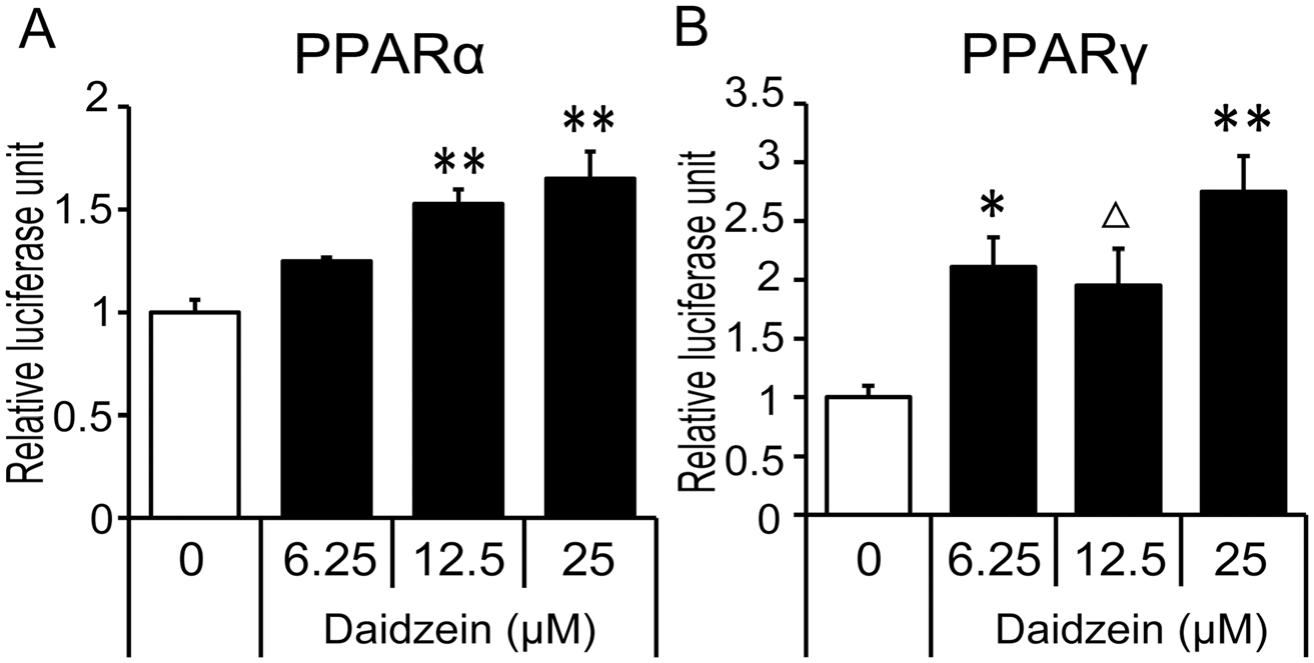
Effect of daidzein on PPAR-α and PPAR-γ activity in HEK293T cells. HEK293T cells were transfected with PPRE-containing reporter plasmid and (A) PPAR-α or (B) PPAR-γ expression plasmids, and then treated with the indicated concentrations (6.25–25 μM) of daidzein for 24 h. Luciferase activity was measured and expressed as a fold induction, that was corrected for transfection efficiency using renilla luciferase activity. Values are expressed as the fold change compared with the vehicle control that was arbitrarily set to 1. Δ, *p*<0.1; *, *p*<0.05; **, *p*<0.01 versus non-treated control.

To examine the involvement of PPARs in the anti-inflammatory action of daidzein, we tested the potential of daidzein to activate PPAR-α and-γ. Direct activation of PPAR-α and-γ by daidzein was confirmed by a luciferase reporter assay. In HEK293T cells, daidzein significantly increased PPAR-α transcriptional activity in a concentration-dependent manner (Fig 3A). Although an obvious dose-dependency was not observed in PPAR-γ transcriptional activity, daidzein also significantly increased PPAR-γ transcriptional activity over a similar range of concentrations at which daidzein enhanced PPAR-α transcriptional activity, with a maximum increase at 25 μM (Fig 3B). As we previously found that concentrations of daidzein above 50 μM induce cell toxicity [21], we used 25 μM daidzein as a maximum dose to activate PPAR-α and-γ in further studies.

### Inhibition of palmitate- and hypertrophied 3T3-L1 conditioned medium induced inflammation by daidzein in RAW264 macrophages

**Fig 4:**
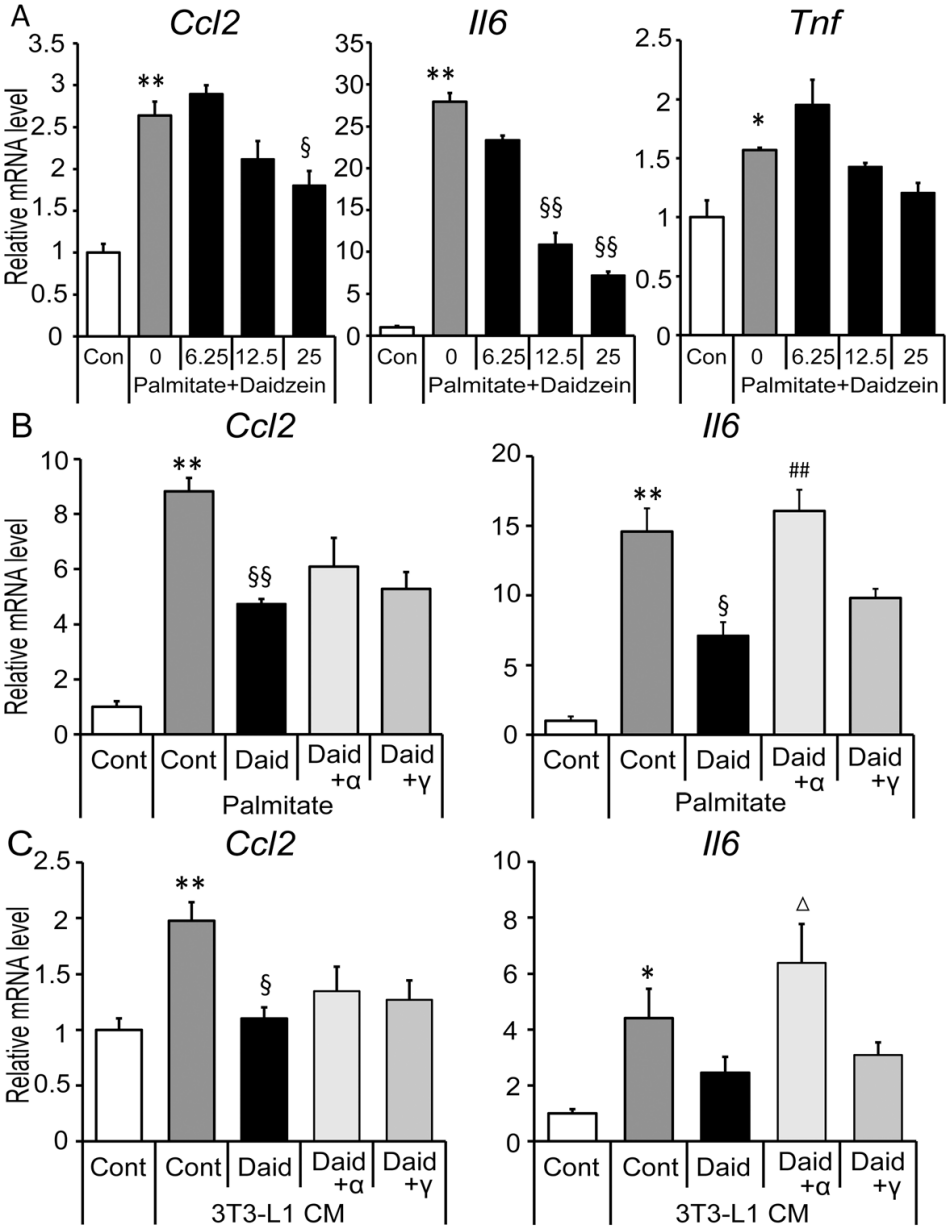
Effects of daidzein via PPAR-α/γ inhibition in palmitate- and CM- treated macrophages. (A) RAW264 macrophages were cultured in the serum-free medium with the indicated concentrations (6.25–25 μM) of daidzein for 1 h, then stimulated with 400 μM palmitate for 4 h. (B) Cells were cultured in serum-free medium with DMSO (Cont), 25 μM of daidzein (Daid), Daid + 10 μM of GW6471 (Daid+α) or Daid + 10 μM of GW9662 (Daid+γ) for 1 h, then 400 μM palmitate was added and cells were incubated for another 4 h. (C) Cells were cultured in CM derived from hypertrophied adipocytes with Daid, Daid+α or Daid+γ for 24 h. Messenger RNA levels of each gene were quantified by real-time PCR and normalized to β-actin expression. Values are expressed as the fold change compared with the vehicle control that was arbitrarily set to 1. *, *p*<0.05; **, *p*<0.01 versus Cont. §, *p*<0.05; §§, *p*<0.01 versus palmitate Cont or CM Cont. Δ, *p*<0.1; ##, *p*<0.01 versus palmitate Daid or CM Daid.

Saturated fatty acids (SFA) like palmitate secreted from adipocytes play a causative role in initiation of adipose tissue inflammation by activating macrophages in a paracrine manner [25]. In the present study, increased expression of *Ccl2* and *Il6* was confirmed in RAW264 macrophages by treatment with 400 μM palmitate and suppressed by daidzein in a dose dependent manner (Fig 4A and 4B). In contrast, *Tnf* enhancement by palmitate was slight and daidzein had no significant effect on *Tnf* gene expression (Fig 4A). Expression of *Ccl2* and *Il6* was also enhanced by CM obtained from hypertrophied 3T3-L1 in which excess of adipocyte-derived SFA triggered inflammatory changes [7], and this enhancement was suppressed by 25 μM daidzein (Fig 4C). To investigate whether PPAR-α and -γ contribute to the inhibition of these genes’ expression by daidzein, we used specific inhibitors for each PPAR. GW6471, a specific inhibitor for PPAR-α, eliminated the reduction of *Il6* expression by daidzein (Fig 4B and 4C), whereas GW9662, a specific inhibitor for PPAR-γ, did not affect the daidzein-mediated decrease of *Il6* expression. Neither of the inhibitors had an effect on the reduction of *Ccl2* gene expression by daidzein (Fig 4B and 4C).

### Regulation of JNK phosphorylation by daidzein in RAW264 macrophages

**Fig 5:**
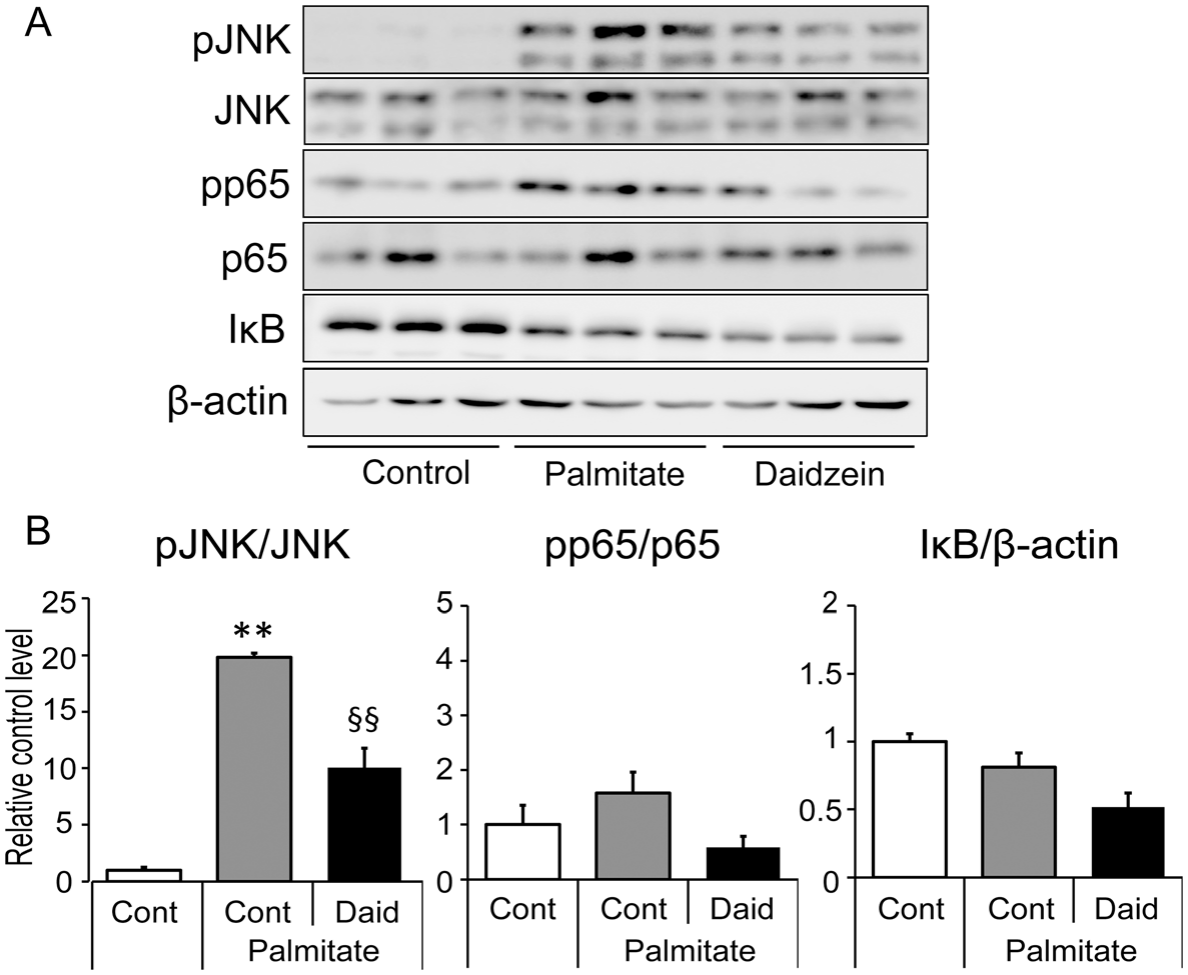
Effect of daidzein on palmitate-induced phosphorylation of JNK and p65 in RAW264 macrophages. RAW264 macrophages were cultured in serum-free medium with (Daid) or without daidzein (Cont) for 1 h. Palmitate was added and cells were incubated for another 4 h. (A) Cells lysates were subjected to immunoblot analysis using specific antibodies. A representative immunoblot from two separate experiments with similar results is shown. (B) Densitometric analysis of protein bands in the western blots was done using Image J software. Optical density for each phosphorylated protein was calculated and normalized with the value of each total protein. **, *p*<0.01 versus Cont. §§, *p*<0.01 versus palmitate Cont.

Previous experimental models suggested that SFA-induced inflammatory changes in the interaction between adipocytes and macrophages via nuclear factor-kappa B (NF-κB)- and Jun-N-terminal kinase (JNK) pathways [26, 27]. Therefore, we next investigated whether NF-κB- or JNK- dependent pathways were involved in the effects of daidzein on regulating SFA-induced inflammation. Intriguingly, palmitate significantly activated JNK, that reached a maximal activation after four hours of treatment, whereas neither significant phosphorylation of NF-κB (p65) nor change of IκB amount was observed by palmitate after four hours of treatment (Fig 5). Pretreatment of macrophages with 25 μM daidzein significantly inhibited this palmitate-induced activation of JNK (Fig 5).

## Discussion

Obesity-induced adipose inflammation by paracrine interactions between adipocytes and adipose-infiltrating macrophages plays a causative role in insulin resistance in obesity [3, 28], and is characterized by abnormal secretion of pro-inflammatory cytokines in white adipose tissues [28, 29]. *In vitro*, co-culture of differentiated 3T3-L1 adipocytes and RAW264 macrophages is used as the model of adipose inflammation in which pro-inflammatory cytokine genes and proteins such as CCL2, IL-6 and TNF-α are significantly upregulated [7, 30]. Several polyphenols were reported to improve inflammatory change in the co-culture model [31–34], however, soy isoflavones have not been tested. Thus, we examined the anti-inflammatory property of daidzein in this model. Treatment with daidzein significantly decreased the elevated levels of *Ccl2* and *Il6* expression seen following co-culture although they did not affect *Tnf* expression in this experimental setting. Previous evidence indicates that CCL2 plays a crucial role in regulating adipose macrophage recruitment and activation [35, 36]. Concomitant with our previous findings that daidzein administration in obese mice resulted in significant decrease of the number of adipose macrophages [21], our present data suggests that daidzein might regulate the macrophage infiltration by inhibiting CCL2 levels in obese tissues. IL-6 is another factor that might be closely related to adipose inflammation and insulin resistance [29, 37]. IL-6 is elevated in obese subjects with insulin resistance and directly causes adipose insulin resistance [38–40]. Therefore, daidzein might improve adipose insulin sensitivity by inhibiting adipose *Il6* expression and consequently reducing expression of the encoded protein.

Although *Ccl2* and *Il6* genes are expressed in both 3T3-L1 adipocytes and RAW264 macrophages, our study suggested that regulatory effects of daidzein on these genes’ expression was less effective in adipocytes in transwell co-culture setting (Fig 2) in which RNA samples were isolated from adipocytes. In contrast, continuous treatment with daidzein protected co-culture-induced reduction of adiponectin, an adipose specific anti-inflammatory cytokine, via a PPAR-γ dependent pathway. Such induction of adiponectin expression by daidzein was not observed in the contact co-culture setting, suggesting that a long duration of daidzein pretreatment was needed to induce adiponectin expression in these cells. To elucidate the anti-inflammatory mechanisms induced by daidzein, especially on *Ccl2* and *Il6* expression, further investigation was conducted with a focus on macrophages. We examined the involvement of both PPAR-α and -γ, that have emerged as key regulators of adipose inflammation [19, 20, 41]. Our data confirmed that daidzein had potential as a dual activator for PPARα/γ, and suppressed the enhancement of *Ccl2* and *Il6* expression induced by palmitate or adipocyte-CM in RAW264 macrophages at the concentration at which it acts as a PPARα/γ activator. In contrast, palmitate had less effect on *Tnf* expression, and consistent with the findings in the co-culture experiment, no significant suppression of *Tnf* induction was observed in the daidzein-treated cells, suggesting a minimal effect of daidzein on *Tnf* expression.

Regarding the involvement of PPARs, a PPAR-α specific inhibitor only abolished this suppressive effect of daidzein on the expression of *Il6*, and the PPAR-γ inhibitor had no effect. Concomitant with the previous report showing that a PPAR-α agonist but not a PPAR-γ agonist reduced IL-6 production and mRNA expression in LPS-stimulated RAW264.7 macrophages [22], our present study indicated that daidzein could attenuate the inflammatory *Il6* expression via PPAR-α activation. Unfortunately, in RAW264 macrophages, IL-6 was too low to be determined by ELISA even when the cells were stimulated by palmitate (data not shown); therefore neither the suppressive effect of daidzein nor the involvement of PPARs on IL-6 production was confirmed in the present study.

SFA secreted from hypertrophied adipocytes plays a causative role in the vicious cycle between adipocytes and macrophages that develops obesity-induced inflammation and insulin resistance [7]. In this SFA-induced inflammation, the NF-κB and JNK-dependent pathways are considered to play a critical role [26, 30]. To characterize further the mechanism of the inhibitory effect of daidzein on inflammatory changes, we analyzed the effect of daidzein on the activation of the NF-κB and JNK pathways. In the present study, palmitate activated JNK phosphorylation and daidzein significantly suppressed this activation of JNK. In contrast, activation of the NF-κB pathway was minimal and no significant suppression was observed by daidzein treatment. This finding was supported by data from Suganami *et al*. [30] who demonstrated that palmitate was capable of inducing *Tnf* expression through the NF-κB pathway in macrophages with much less potency than LPS.

Previous reports suggested that JNK plays crucial roles in CCL2 expression in both adipocytes and macrophages [27,42]. Additionally, a previous study using CM-stimulated macrophages reported that CCL2 production was suppressed by luteolin, a flavone, through inhibition of JNK activation but not of NF-κB activation [32]. It is well accepted that phosphorylated JNK activates the oncoprotein c-Jun, which forms part of the activator protein-1 (AP-1) transcription factor, thereby enhancing the expression of various pro-inflammatory genes. Concomitant with these findings, our present study suggests the anti-inflammatory effect of daidzein on SFA-stimulated macrophages is dependent in part on JNK and AP-1, especially with respect to *Ccl2* expression. The JNK pathway is also considered to be involved in *Tnf* expression; however, it was suggested that NF-κB is a more potent and independent regulator of *Tnf* expression [30, 43]. No significant changes were observed in *Tnf* expression in the present study, possibly because of the minimal effect of daidzein on the NF-κB pathway.

**Fig 6:**
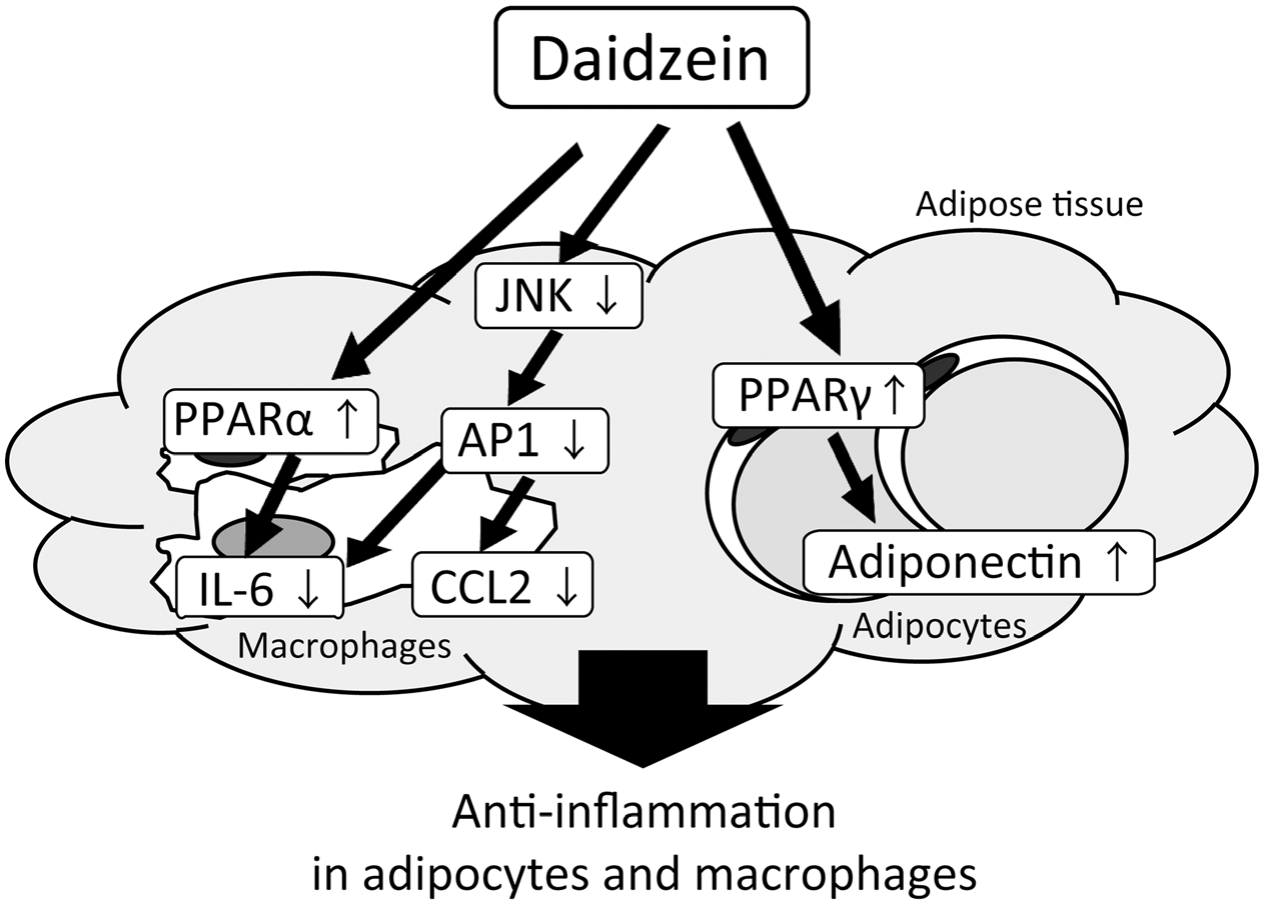
Putative mechanisms for the anti-inflammatory effect of daidzein on adipocyte-macrophage co-cultures.

In conclusion, this study provides *in vitro* evidence that daidzein regulates the expression and secretion of pro-inflammatory cytokines such as CCL2 and IL-6 in adipocyte-macrophage co-cultures via multiple pathways in which PPAR-α, PPAR-γ and JNK are involved (the putative mechanism is summarized in Fig 6). The anti-inflammatory mechanism of this isoflavone in which PPAR-α is involved is a unique and novel finding. Recently, an interesting study was reported by Velpen *et al*. that soy isoflavone supplementation predominantly containing daidzein and daidzin (daidzein glucoside) resulted in caloric restriction–like and anti-inflammatory gene expression profiles in adipose tissues of postmenopausal women [44]. In humans, the serum level of daidzein following the single consumption of tens of grams of soybean nuts, as measured by the area under the curve, reached approximately 20 μmol/L•h, similar to the concentration we used in vitro (25 μM) [45]. Therefore, daidzein or soy consumption could be a therapeutic strategy for suppressing the vicious cycle of chronic inflammation in obese adipose tissue, and might improve obesity-related insulin resistance.

## Supporting Information

S1 Fig Protocol for the co-culture system of 3T3-L1 adipocytes and RAW264 macrophages. (TIF)

S1 Table Sequences of primers for each gene. (PDF)

S2 Table Antibodies used in this study. (PDF)
