# Supplementary figures and images for: The Dietary Isoflavone Daidzein Reduces Expression of Pro-Inflammatory Genes through PPARα/γ and JNK Pathways in Adipocyte and Macrophage Co-Cultures
Source: PLoS One. 2016 Feb 22;11(2):e0149676. doi: 10.1371/journal.pone.0149676 (PMC4763373; doi:10.1371/journal.pone.0149676)

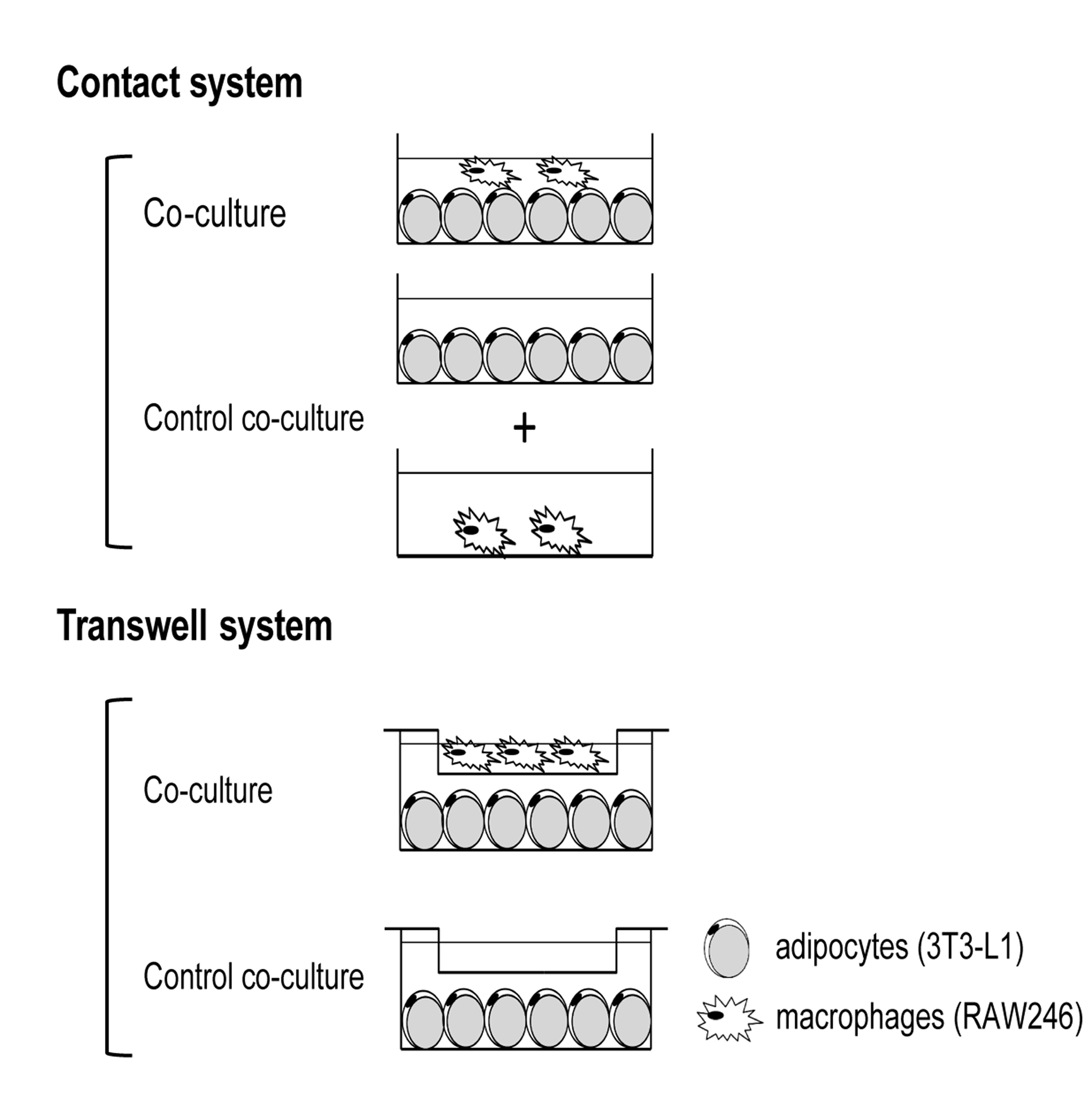

Supplement: S1 Fig — (TIF) [file pone.0149676.s001.tif]
