# Supplementary material for: The Dietary Isoflavone Daidzein Reduces Expression of Pro-Inflammatory Genes through PPARα/γ and JNK Pathways in Adipocyte and Macrophage Co-Cultures
Source: PLoS One. 2016 Feb 22;11(2):e0149676. doi: 10.1371/journal.pone.0149676 (PMC4763373; doi:10.1371/journal.pone.0149676)
Supplement: S1 Table — (PDF) [file pone.0149676.s002.pdf]

Supporting Table 1. Sequences of primers for each gene

Target (Accession #)	Forward	Reverse
Adipoq (Adiponectin) (NM_009605.4)	ATGGCAGAGATGGCACTCCT	CCTTCAGCTCCTGTCATTCCA
Ccl2 (NM_011333.3)	CCACTCACCTGCTGCTACTCAT	TGGTGATCCTCTTGTAGCTCTCC
Il6 (NM_031168.1)	ACAACCACGGCCTTCCCTACTT	CACGATTTCCCAGAGAACATGTG
Tnf (NM_013693.3)	ACCCTCACACTCAGATCATCTTC	TGGTGGTTTGCTACGACGT
Actb (β-Actin) (NM_007393.3)	CTAAGGCCAACCGTGAAAAG	ACCAGAGGCATACAGGGACA
