# Supplementary material for: The Dietary Isoflavone Daidzein Reduces Expression of Pro-Inflammatory Genes through PPARα/γ and JNK Pathways in Adipocyte and Macrophage Co-Cultures
Source: PLoS One. 2016 Feb 22;11(2):e0149676. doi: 10.1371/journal.pone.0149676 (PMC4763373; doi:10.1371/journal.pone.0149676)
Supplement: S2 Table — (PDF) [file pone.0149676.s003.pdf]

Supporting Table 2. Antibodies used in this study

Primary antibodies

antibody	mono/poly	host species	supplier	catalogue number	dilution
JNK	polyclonal	rabbit	Santa Cruz Biotechnology	SC-571	1:3000
pJNK	monoclonal		Cell Signaling	#4668	1:3000
p65	polyclonal		Santa Cruz Biotechnology	SC-372	1:3000
pp65	monoclonal		Cell Signaling	#3033	1:3000
IκB	polyclonal		Santa Cruz Biotechnology	SC-371	1:3000
β-actin	monoclonal	mouse	Santa Cruz Biotechnology	SC-47778	1:3000

Secondary antibodies

antibody	mono/poly	host species	supplier	catalogue number	dilution
HRP-linked anti-rabbit IgG	polyclonal	goat	Cell signaling	#7074	1:7000
HRP-linked anti-mouse IgG	polyclonal	horse	Cell Signaling	#7076	1:10000
